# Atypical clinical variants of Alzheimer’s disease: are they really atypical?

**DOI:** 10.3389/fnins.2024.1352822

**Published:** 2024-02-28

**Authors:** Jennifer L. Whitwell

**Affiliations:** Department of Radiology, Mayo Clinic, Rochester, MN, United States

**Keywords:** Alzheimer’s disease, posterior cortical atrophy, logopenic aphasia, corticobasal, neuroimaging, biomarkers, tau PET, TDP-43

## Abstract

Alzheimer’s disease (AD) is a neuropathological disorder defined by the deposition of the proteins, tau and β-amyloid. Alzheimer’s disease is commonly thought of as a disease of the elderly that is associated with episodic memory loss. However, the very first patient described with AD was in her 50’s with impairments in multiple cognitive domains. It is now clear that AD can present with multiple different non-amnestic clinical variants which have been labeled as atypical variants of AD. Instead of these variants of AD being considered “atypical,” I propose that they provide an excellent disease model of AD and reflect the true clinical heterogeneity of AD. The atypical variants of AD usually have a relatively young age at onset, and they show striking cortical tau deposition on molecular PET imaging which relates strongly with patterns of neurodegeneration and clinical outcomes. In contrast, elderly patients with AD show less tau deposition on PET, and neuroimaging and clinical outcomes are confounded by other age-related pathologies, including TDP-43 and vascular pathology. There is also considerable clinical and anatomical heterogeneity across atypical and young-onset amnestic variants of AD which reflects the fact that AD is a disease that causes impairments in multiple cognitive domains. Future studies should focus on careful characterization of cognitive impairment in AD and consider the full clinical spectrum of AD, including atypical AD, in the design of research studies investigating disease mechanisms in AD and clinical treatment trials, particularly with therapeutics targeting tau.

## Introduction

Alzheimer’s disease (AD) was first described in 1906 with the description of a patient in her 50’s who developed memory impairment, language problems and changes in behavior and was found to have neurofibrillary tangles and senile plaques in her brain at autopsy (Alzheimer, 1907). The abnormal proteins in these brain lesions were later identified as tau (Kidd, 1963; Grundke-Iqbal et al., 1986) and β-amyloid (Glenner and Wong, 1984). This initial case was labeled as a presenile dementia, but the concept of AD was later expanded to include senile dementia affecting patients over age 65 years (also known as late-onset AD). In the 1980’s diagnostic criteria for AD were published that defined probable Alzheimer’s dementia as a predominantly amnestic disorder in which other cognitive domains can become affected (McKhann et al., 1984). Most studies in the 1990’s focused on characterizing this amnestic-predominant AD with neuroimaging studies highlighting atrophy of the hippocampus (Seab et al., 1988; Jack et al., 1992) and whole brain (Fox et al., 1996), and neuropathological findings showing that tau pathology spreads from the transentorhinal region to the hippocampus and then the lateral temporal and cortical regions (Braak and Braak, 1991). It was also shown that the prevalence of amnestic AD increases with age (Katzman, 1976) and, hence the concept of AD being an amnestic disease affecting the elderly became the generally held view of AD. However, during the 1980’s and early 90’s, cases were reported that had pathological evidence of AD but who presented with non-amnestic features, including visual symptoms (Hof et al., 1989; Levine et al., 1993; Ross et al., 1996), alien-hand phenomenon (Ball et al., 1993), aphasia (Pogacar and Williams, 1976; Green et al., 1990) and frontal/executive dysfunction (Johnson et al., 1999; Back-Madruga et al., 2002). Small case series were subsequently reported showing that the clinical presentation in AD was heterogeneous (Neary et al., 1986; Price et al., 1993; Galton et al., 2000). It was recognized that these different cognitive presentations were associated with different distributions of tau pathology in the brain and different patterns of atrophy (Kanne et al., 1998; Galton et al., 2000), with greater involvement of the cortex and relative sparing of the hippocampus compared to amnestic AD patients (Galton et al., 2000). This pattern of relatively greater tau deposition in the cortex compared to the hippocampus was later defined as the hippocampal-sparing variant of AD which contrasted with limbic-predominant patterns in patients with relatively greater involvement of the hippocampus compared to the cortex (Murray et al., 2011). These patients tend to have a younger age at onset compared to amnestic AD, accounting for at least a third of AD cases with onset under age 65 years (Koedam et al., 2010; Sarto et al., 2022; Tort-Merino et al., 2022), and hence were labeled “atypical AD” (Galton et al., 2000); a term that has persisted over time.

**Figure 1:**
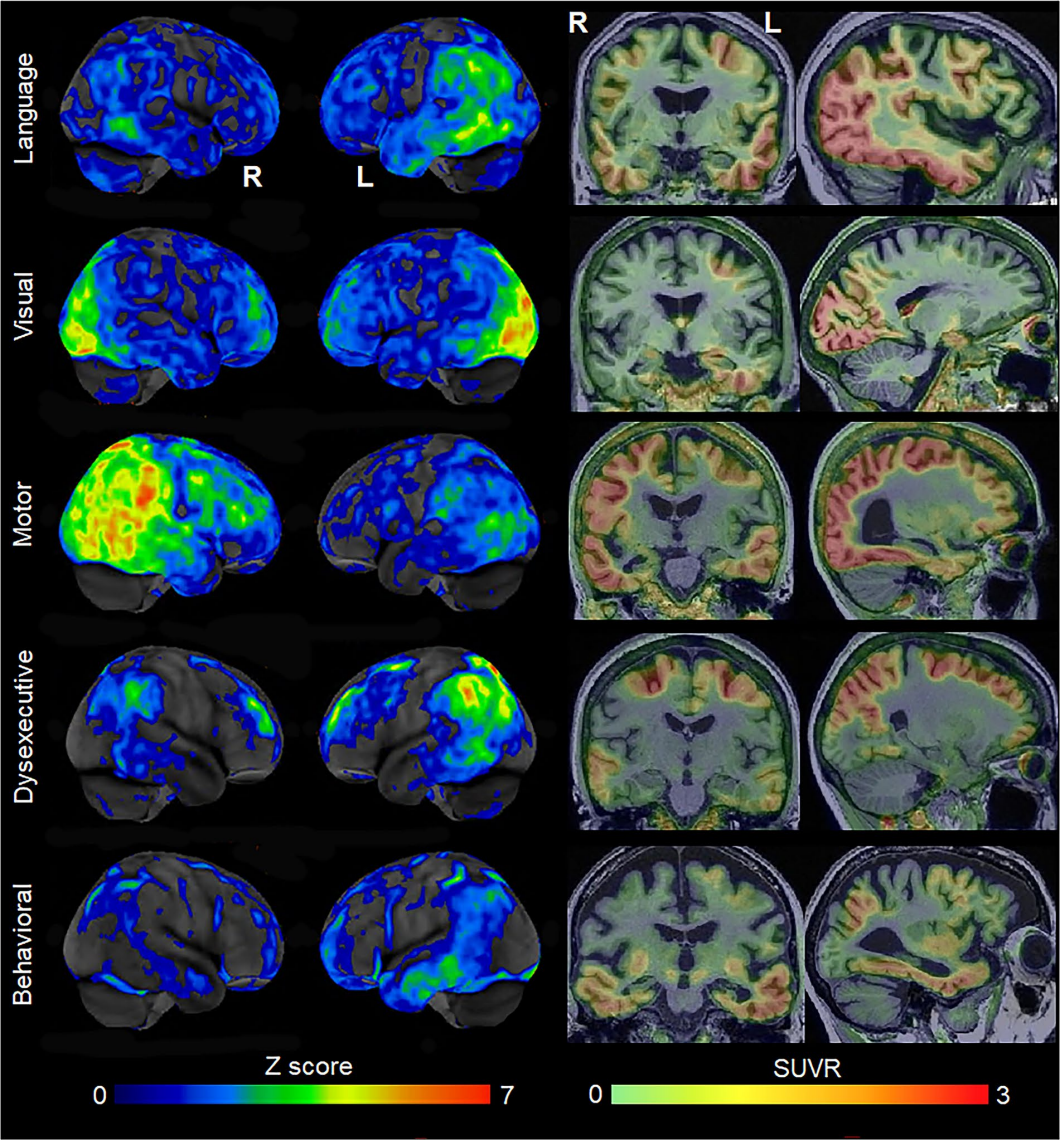
Patterns of FDG-PET hypometabolism and flortaucipir PET uptake in individual patients with different variants of atypical AD. The FDG-PET images are shown as CortexID Z score images of hypometabolism compared to age-matched controls. The flortaucipir PET images are shown as standardized uptake value ratio (SUVR) images referenced to the cerebellar crus grey matter. The language variant of AD shows left temporoparietal hypometabolism and flortaucipir uptake; the visual variant shows abnormalities predominantly in the occipital lobe; the motor variant shows abnormalities in the posterior temporal, parietal and frontal lobes, predominantly on the right; the dysexecutive variant shows left-sided abnormalities predominantly in the superior parietal and frontal lobes; the behavioral variant showed abnormalities in the temporal lobes, with milder changes in the parietal and frontal lobe.

Since the early 2000’s, there has been increasing recognition and characterization of atypical variants of AD. The visual variant of AD is synonymous with the diagnosis of posterior cortical atrophy (Tang-Wai et al., 2004; Crutch et al., 2017), with patients presenting with visuospatial and perceptual difficulties and other posterior cognitive functions, including simultanagnosia, Gerstmann syndrome, oculomotor apraxia, and optic ataxia. There has been characterization of the language variant of AD recognizing that the most common symptoms include anomia with word-finding pauses (Rogalski et al., 2016) and trouble with sentence repetition, with phonological sound errors often observed (Mesulam et al., 2008). Some of these patients can meet criteria for the logopenic variant of primary progressive aphasia (Gorno-Tempini et al., 2011), although in many cases the presence of other cognitive impairments may preclude the diagnosis of primary progressive aphasia (Owens et al., 2018). Alzheimer’s disease pathology has also been observed as the primary pathology in some patients with corticobasal syndrome, with these patients referred to as the motor (or praxic) variant of AD (Graff-Radford et al., 2021). These patients present with asymmetric rigidity, bradykinesia, dystonia, myoclonus, ideomotor apraxia, cortical sensory deficit, and alien limb phenomenon (Armstrong et al., 2013). Alzheimer’s disease can also present with changes in behavior and personality, mimicking the behavioral variant of frontotemporal dementia (Forman et al., 2006; Beach et al., 2012). These patients have been defined to the behavioral variant of AD (Ossenkoppele et al., 2015, 2022). Lastly, the dysexecutive variant of AD has recently been defined where patients have predominant problems with core executive functions of working memory, cognitive flexibility, and cognitive inhibitory control (Townley et al., 2020). These atypical variants of AD are all associated with neurodegeneration of the cortex, with differing regional patterns associated with each variant (Figure 1). The visual variant of AD is associated with atrophy and hypometabolism of the parietal and occipital lobes (Whitwell et al., 2007), the language variant with involvement of the left temporoparietal cortex (Gorno-Tempini et al., 2004), the motor variant with involvement of the frontal, posterior temporal and parietal lobes (Josephs et al., 2010), the behavioral variant with involvement of the temporoparietal and frontal cortex (Ossenkoppele et al., 2022), and the dysexecutive variant with involvement of frontoparietal cortex (Townley et al., 2020). The atypical variants of AD tend to show different patterns of atrophy than amnestic AD, with relative sparing of the hippocampus (Josephs et al., 2020b) and different patterns of cortical involvement, at least early in the disease (Phillips et al., 2018), although overlap has been observed, particularly between the behavioral variant of AD and amnestic AD (Ossenkoppele et al., 2015; Singleton et al., 2020; Therriault et al., 2021). The clinicopathological concordance of these clinical phenotypes with AD varies. A large proportion of patients presenting with the features of the visual and language variants of AD have underlying AD, approximately 30% of corticobasal syndrome have AD (Hu et al., 2009; Ling et al., 2010; Lee et al., 2011; Boyd et al., 2014; Ouchi et al., 2014; Koga et al., 2022; Shir et al., 2023), and AD is relatively rare (<10%) in patients that present with behavior and personality change (Kertesz et al., 2005). However, molecular PET ligands that can detect β-amyloid and AD-tau deposition in the brain antemortem have made the diagnosis of AD in patients with these clinical presentations much easier during life. Evidence of deposition on both β-amyloid and tau PET scans provides a biomarker diagnosis of AD (Jack et al., 2018). Most patients diagnosed with the visual and language variants of AD at specialist centers have biomarker evidence for AD (Singh et al., 2022).

Despite detailed clinical characterization of these atypical AD variants, the advent of molecular PET to aid diagnosis, and the fact that these atypical variants of AD have been included in recent clinical diagnostic criteria for AD (McKhann et al., 2011; Dubois et al., 2021), there is still a lack of consideration of these patients in the large majority of the AD literature with these variants considered rare compared to the “typical” amnestic presentation of AD. Importantly, these patients are also not commonly considered in clinical treatment trials for AD which often still require amnestic impairment. It is, therefore, difficult to know how currently available AD therapeutics may perform in these populations, even though they are eligible for treatment. I propose that these atypical variants of AD provide the ideal disease model of AD and should be the focus of future research into AD disease mechanisms and therapies. The fact that these phenotypes have been labeled with the term “atypical” has likely done them a disservice and minimized their perceived importance and value to the field.

## Strong relationship between tau, neurodegeneration, and clinical symptoms

**Figure 2:**
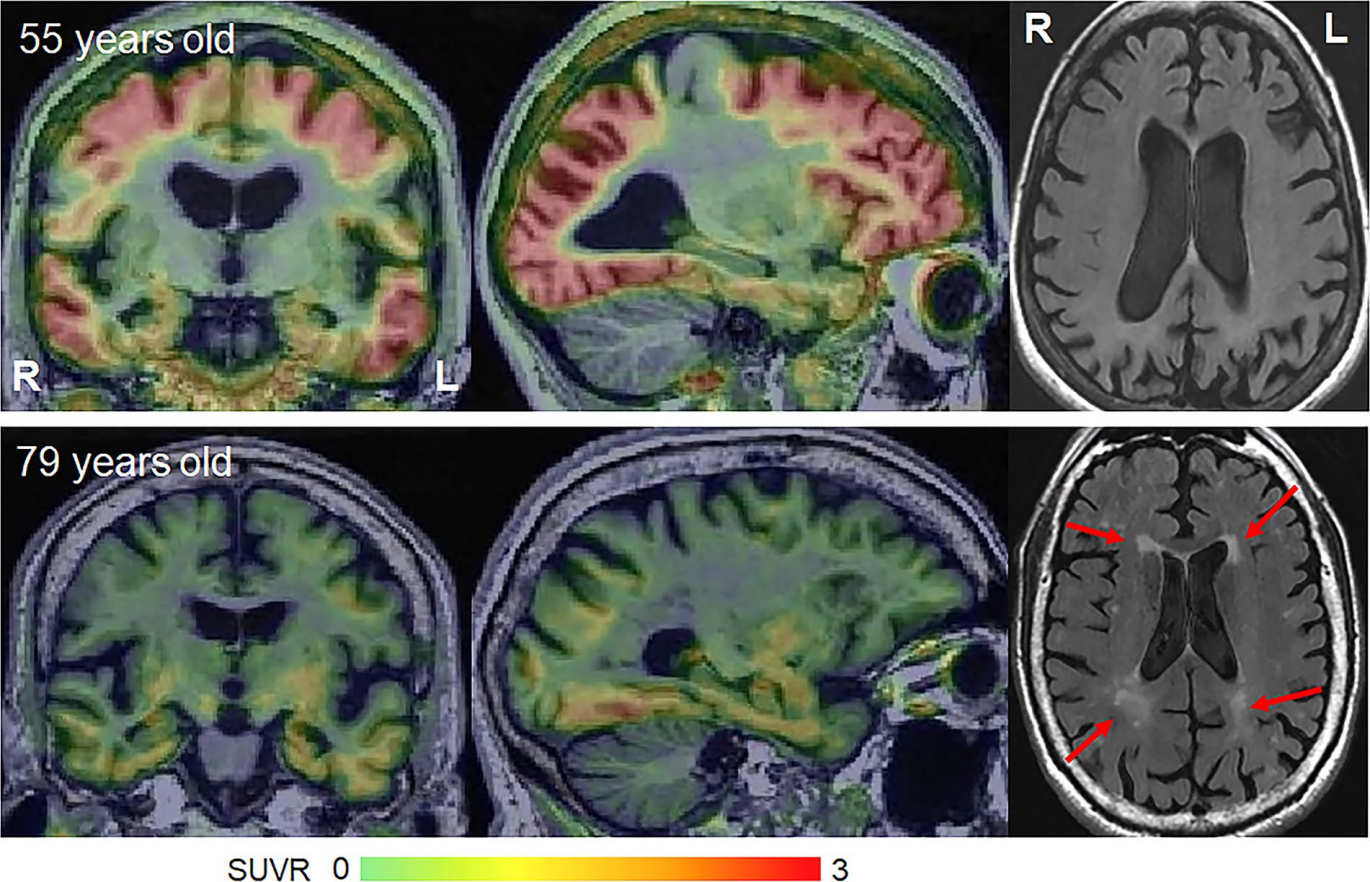
Flortaucipir PET uptake and white matter hyperintensities on FLAIR in young-onset and late-onset amnestic AD patients. The flortaucipir PET images are shown as standardized uptake value ratio (SUVR) images referenced to the cerebellar crus grey matter. The young-onset patient shows widespread flortaucipir uptake with no WMHs, while the late-onset patient shows milder flortaucipir uptake and moderate WMHs.

Molecular PET findings in the atypical variants of AD have been well characterized over the last few years. In β-amyloid PET positive atypical AD patients, striking patterns of cortical tau deposition are observed using tau PET ligands such as ^18^F-flortaucipir [formally known as AV-1451 (Xia et al., 2017)]. Patterns of uptake differ across the AD variants, largely matching the patterns of atrophy observed in each variant (Ossenkoppele et al., 2016; Sintini et al., 2020), although overlap across variants is observed (Figure 1). The patterns of tau uptake also concur with pathological studies showing different regional distributions of neurofibrillary tangles across atypical and amnestic variants of AD (Gefen et al., 2012; Josephs et al., 2013; Petersen et al., 2019; Martersteck et al., 2022). The degree of cortical tau deposition in AD is strongly related to age, with greater deposition observed in younger patients (Whitwell et al., 2018a, 2019; La Joie et al., 2021; Tanner et al., 2022). Among younger AD patients, those with atypical presentations tend to show similar degrees of cortical tau uptake on PET compared to amnestic AD, but lower uptake in hippocampus and entorhinal regions (Whitwell et al., 2018a; Josephs et al., 2020b). In these young AD patients, tau uptake is strongly regionally related to volume loss and hypometabolism on FDG-PET (Whitwell et al., 2018b; Sintini et al., 2019), as well as degeneration of a network of white matter tracts (Sintini et al., 2019). These findings support the fact that tau is the driving force behind neurodegeneration in atypical AD, as well as young-onset amnestic AD. Tau uptake, volume loss and hypometabolism are also strongly associated with clinical outcomes in these patients (Tanner et al., 2022). However, older amnestic AD patients over the age of 65 years can show very little cortical or medial temporal uptake on PET (Whitwell et al., 2018a; Figure 2) and the relationship between tau uptake and volume loss breaks down, with volume loss of the medial temporal lobe occurring in the absence of appreciable tau uptake (Josephs et al., 2020b), suggesting that other factors are contributing to this volume loss in elderly AD patients. Hence, it would be advisable to recruit patients with atypical AD or young-onset amnestic AD in treatment trials of therapeutic agents that target tau since we need a disease model where tau is central to the degenerative process. Furthermore, atypical AD patients show greater change over time in tau accumulation measured on PET compared to amnestic AD (Sintini et al., 2020), and, hence, it would be more feasible to assess longitudinal tau outcome measures in treatment trials in these patients.

## Relative lack of other confounding pathologies

Many additional pathologies can be found in the brains of AD patients that contribute to cognitive decline and neurodegeneration in older patients. One such contributing pathology is the presence of the TAR DNA binding protein of 43 kDa (TDP-43). This protein is one of the major disease proteins in frontotemporal lobar degeneration (Neumann et al., 2006), but abnormal TDP-43 is also observed in many patients with AD pathology (Amador-Ortiz et al., 2007; Josephs et al., 2008; Uryu et al., 2008; Arai et al., 2009; Davidson et al., 2011). The prevalence of TDP-43 increases with age, with TDP-43 observed in almost 70% of AD patients at age 90 years (Carlos et al., 2022). TDP-43 pathology in AD progresses in distinct stages, beginning in the amygdala before spreading to the entorhinal cortex and hippocampus, then to the inferior temporal lobe, basal forebrain, insula, and striatum, followed by the midbrain and finally the frontal lobes (Josephs et al., 2014a, 2016). The presence of abnormal TDP-43 is associated with worse cognition (Josephs et al., 2008, 2014b; Wilson et al., 2013), smaller brain volumes (Josephs et al., 2008, 2014b) and faster rates of atrophy (Josephs et al., 2017), particularly affecting the medial temporal lobes, with worse outcomes associated with a greater burden of TDP-43 and higher TDP-43 stage (Josephs et al., 2014b, 2020a). However, TDP-43 plays a larger role in older patients with AD. The frequency of TDP-43 is relatively low in the hippocampal sparing pathological variant of AD (21%) (Josephs et al., 2015) which is associated with atypical AD, and TDP-43 is observed in <25% of patients aged 65 years and younger (Carlos et al., 2022). In fact, TDP-43 was only observed in 12% of one atypical AD cohort (Bigio et al., 2010) and 12.5% of a young-onset AD cohort (Sarto et al., 2022). Furthermore, there is little evidence that clinical or anatomical features in atypical AD are related to TDP-43 (Sahoo et al., 2018). In older amnestic AD patients, it is likely that TDP-43-related medial temporal atrophy explains the breakdown between volume and tau uptake on PET (Carlos et al., 2023). In other words, some of the medial temporal atrophy observed in these older patients is driven by TDP-43, and not tau.

Vascular pathology is also a major contributor to cognitive impairment. Small vessel disease which manifests on MRI as subcortical infarcts, white matter hyperintensities (WMHs) or subcortical microhemorrhages (microbleeds) is strongly age-related (Scheltens et al., 1992; Whitwell et al., 2015) and is found at autopsy in over 50% of people aged ≥65 years (Vernooij et al., 2008; Hainsworth et al., 2023). In fact, WMHs are observed in 95% of healthy adults over 65 years (Longstreth et al., 1996; Figure 2). Small vessel disease is associated with worse cognition, cerebral brain atrophy and medial temporal atrophy in late onset amnestic AD (Snowdon et al., 1997; Capizzano et al., 2004; Logue et al., 2011; Chen et al., 2022), with small vessel disease providing an additive effect to that of AD pathology (Launer et al., 2008). However, young-onset AD is not associated with vascular risk factors (Carotenuto et al., 2012; Chen et al., 2017) and the burden of small vessel disease, including WMHs, is lower than that observed in late onset amnestic AD (Scheltens et al., 1992; Carotenuto et al., 2012). WMHs are relatively rare in atypical AD, affecting ~15% of patients, and are only observed in atypical AD patients >65 years (Pham et al., 2022). There is, however, some evidence that WMHs may be associated with visuoperceptual impairment in atypical AD (Pham et al., 2022). Cortical and subcortical infarcts are rarely observed in atypical AD (Pham et al., 2022). Lobar microbleeds have been observed in 30–50% of atypical AD patients (Whitwell et al., 2014, 2015; Mendes et al., 2018), although the prevalence appears to be highest in the language variant of AD compared to other atypical variants (Whitwell et al., 2015; Ikeda et al., 2021) and microbleeds are not associated with clinical outcomes (Whitwell et al., 2015). The presence of microbleeds on MRI in atypical AD is associated with moderate–severe underlying cerebral amyloid angiopathy (Buciuc et al., 2021). Hence, small vessel disease appears to play some role in atypical AD, likely in atypical AD patients over age 65, but is not as big a confounder as in late-onset amnestic AD.

## Clinical overlap across atypical AD variants

The atypical AD variants are each characterized by early and predominant impairment in a specific cognitive or behavioral domain, i.e., language, visual, motor, executive or behavior. However, these patients can present with impairments affecting other cognitive domains or develop abnormalities in these other domains over time. Early anomia, reduced verbal fluency, trouble with sentence repetition, and slowed speech rate (i.e., features of the language variant of AD), as well as phonological errors, can occur in patients with the visual variant of AD (Crutch et al., 2013; Tetzloff et al., 2021; Volkmer et al., 2022), and have been observed in 50% of patients (Singh et al., 2023). These language features relate to overlapping neuroanatomical features, particularly with atrophy in the left temporoparietal regions in patients with the visual variant of AD (Wicklund et al., 2013; Tetzloff et al., 2021). Conversely, patients with the language variant of AD commonly (~30%) present with some visual dysfunction, including visuospatial/perceptual deficits (Tippett et al., 2020; Foxe et al., 2021; Tee et al., 2022), simultanagnosia and Gerstmann syndrome (i.e., features of the visual variant of AD), which is related to atrophy and tau deposition developing in the parietal and occipital lobes (Singh et al., 2023). Patients with the language variant of AD can also display episodic memory impairment (Foxe et al., 2021; Tee et al., 2022; Whitwell et al., 2022), executive dyfunction (Basaglia-Pappas et al., 2023), and ideomotor apraxia (Crutch et al., 2013). In fact, marked cognitive deficits outside the domain of language are observed in at least 50% of patients with the language variant of AD (Machulda et al., 2013; Owens et al., 2018). Patients with the motor variant of AD can also present with visuospatial/perceptual deficits, simultanagnosia, visual neglect, acalculia, optic ataxia, and episodic memory loss (Shelley et al., 2009; Lee et al., 2011; Boyd et al., 2014; Shir et al., 2023). Episodic memory loss is also observed in the dysexecutive (Corriveau-Lecavalier et al., 2023) and behavioral variants of AD (Ossenkoppele et al., 2022), with visuospatial deficits observed in some dysexecutive AD patients (Corriveau-Lecavalier et al., 2023). In fact, the degree of episodic memory impairment can be similar across atypical and amnestic variants of AD (Mendez et al., 2019), particularly the behavioral variant of AD (Ossenkoppele et al., 2022), although this may depend on the stage and severity of the patients included and issues with test scores being confounded by other cognitive deficits. These overlapping features often make it difficult to phenotypically classify patients (Wicklund et al., 2013; Fitzpatrick et al., 2019). It appears phenotypic overlap across variants in atypical AD is the rule rather than the exception, with only 25% of atypical AD patients in one series showing a “pure” presentation (Singh et al., 2023). Patterns of clinical decline in atypical AD over time are similarly heterogeneous (Whitwell et al., 2022). Several studies have also shown that early-onset AD is more cognitively heterogeneous than late-onset AD (Koedam et al., 2010; Joubert et al., 2016; Tort-Merino et al., 2022). It is, therefore, evident that there is a phenotypic continuum across young-onset patients, and the clinical distinction between different AD variants may be somewhat artificial. It might be time to stop thinking of these clinical variants as “atypical” and instead think of them as AD which presents with cognitive impairment that affects multiple domains. I would also argue that there lacks rationale for focusing on just one clinical variant (i.e., amnestic AD) for therapeutic treatment trials, and that all variants of AD should be included to capture the true clinical spectrum of AD. The high degree of clinical overlap in these AD patients should also be considered for appropriate tailoring of patient management strategies (Volkmer et al., 2022).

## Discussion

Research over the past two decades has characterized the clinical, neuroanatomical, and molecular biology of atypical AD. It has become clear that AD with a relatively young age at onset is different from AD observed in the elderly, with neurodegeneration and cognition strongly driven by tau deposition in young patients while contributions of TDP-43 and vascular pathology are important components to disease in older patients. Atypical AD provides a clear disease model of AD driven by tau. Young-onset patients show considerable heterogeneity with clinical and neuroanatomical overlap across variants and there is evidence that this heterogeneity relates to the topographic distribution of tau throughout the cortex. This heterogeneity, in my opinion, reflects the true clinical phenotype of AD and challenges the notion of AD as an amnestic disease. In fact, the amnestic presentation in many older patients could be driven by TDP-43 and other age-related pathologies, and not AD. As such, it is unclear whether pharmacological treatments tested in elderly amnestic AD patients will generalize to AD observed in younger and atypical AD patients, and clinical treatment trials should recruit atypical AD patients. It might be time to revise our views on atypical AD and not consider it so “atypical.”

There are, however, several challenges related to the study and assessment of atypical AD that will need to be overcome. Clinical diagnosis and phenotyping can be challenging given the high degree of phenotypic overlap across AD variants and overlap with other neurodegenerative diseases. Increasing awareness of the atypical presentations of AD will be crucial to avoid early misdiagnosis (Balasa et al., 2011; Graff-Radford et al., 2021) and to improve prevalence estimates of these presentations of AD (Graff-Radford et al., 2021). Molecular PET, cerebrospinal fluid biomarkers (Wellington et al., 2018; Pillai et al., 2019) and, likely, blood plasma biomarkers, will be crucial to confirm the presence of AD. Clinical heterogeneity also makes it challenging to develop clinical outcome measures that could be used as disease biomarkers across all patients and variants, and hence more generalizable clinical measures are needed. Neuroimaging biomarkers (Whitwell et al., 2011) or fluid biomarkers (Wellington et al., 2018; Pillai et al., 2019) may be more effective outcome measures. Neuroimaging biomarkers that are commonly utilized, however, focus on assessing medial temporal regions appropriate in late-onset AD and it will be important to develop biomarkers that can detect changes across atypical and amnestic AD or multiple biomarkers may be needed. There is also a lack of knowledge regarding the prevalence and characteristics of atypical AD in minority populations, with most studies assessing white non-Hispanic patients. Future studies should focus on careful characterization of cognitive impairment in AD and consider the full clinical spectrum of AD in the design of research studies and clinical treatment trials.

## Data availability statement

The original contributions presented in the study are included in the article/supplementary material, further inquiries can be directed to the corresponding author.

## Author contributions

JW: Conceptualization, Data curation, Funding acquisition, Writing – original draft.

## References

[ref1] Alzheimer A. (1907). Uber eine eignartige Erkrankung der Hirnrinde (about a peculiar disease of the cerebral cortex; English translation Alz dis ass dis 1:3–8, 1987). Allgemeine Z Psychiatrie Psychisch Gerichtliche Medizin 64, 146–148.

[ref2] Amador-Ortiz C. Lin W. L. Ahmed Z. Personett D. Davies P. Duara R.. (2007). TDP-43 immunoreactivity in hippocampal sclerosis and Alzheimer's disease. Ann. Neurol. 61, 435–445. doi: 10.1002/ana.21154, PMID: 17469117 PMC2677204

[ref3] Arai T. Mackenzie I. R. Hasegawa M. Nonoka T. Niizato K. Tsuchiya K.. (2009). Phosphorylated TDP-43 in Alzheimer's disease and dementia with Lewy bodies. Acta Neuropathol. 117, 125–136. doi: 10.1007/s00401-008-0480-1 19139911

[ref4] Armstrong M. J. Litvan I. Lang A. E. Bak T. H. Bhatia K. P. Borroni B.. (2013). Criteria for the diagnosis of corticobasal degeneration. Neurology 80, 496–503. doi: 10.1212/WNL.0b013e31827f0fd1, PMID: 23359374 PMC3590050

[ref5] Back-Madruga C. Boone K. B. Briere J. Cummings J. McPherson S. Fairbanks L.. (2002). Functional ability in executive variant Alzheimer's disease and typical Alzheimer's disease. Clin. Neuropsychol. 16, 331–340. doi: 10.1076/clin.16.3.331.13846, PMID: 12607146

[ref6] Balasa M. Gelpi E. Antonell A. Rey M. J. Sánchez-Valle R. Molinuevo J. L.. (2011). Clinical features and APOE genotype of pathologically proven early-onset Alzheimer disease. Neurology 76, 1720–1725. doi: 10.1212/WNL.0b013e31821a44dd 21576687

[ref7] Ball J. A. Lantos P. L. Jackson M. Marsden C. D. Scadding J. W. Rossor M. N. (1993). Alien hand sign in association with Alzheimer's histopathology. J. Neurol. Neurosurg. Psychiatry 56, 1020–1023. doi: 10.1136/jnnp.56.9.1020, PMID: 8410026 PMC489742

[ref8] Basaglia-Pappas S. Laurent B. Getenet J. C. Boulangé A. Rendón de laCruz A. Simoes Loureiro I.. (2023). Executive profile of the Logopenic variant of primary progressive aphasia: comparison with the semantic and non-fluent variants and Alzheimer's disease. Brain Sci. 13:13. doi: 10.3390/brainsci13030406 PMC10046635 36979216

[ref9] Beach T. G. Monsell S. E. Phillips L. E. Kukull W. (2012). Accuracy of the clinical diagnosis of Alzheimer disease at National Institute on Aging Alzheimer disease centers, 2005–2010. J. Neuropathol. Exp. Neurol. 71, 266–273. doi: 10.1097/NEN.0b013e31824b211b, PMID: 22437338 PMC3331862

[ref10] Bigio E. H. Mishra M. Hatanpaa K. J. White C. L. III Johnson N. Rademaker A.. (2010). TDP-43 pathology in primary progressive aphasia and frontotemporal dementia with pathologic Alzheimer disease. Acta Neuropathol. 120, 43–54. doi: 10.1007/s00401-010-0681-2, PMID: 20361198 PMC2903745

[ref11] Boyd C. D. Tierney M. Wassermann E. M. Spina S. Oblak A. L. Ghetti B.. (2014). Visuoperception test predicts pathologic diagnosis of Alzheimer disease in corticobasal syndrome. Neurology 83, 510–519. doi: 10.1212/WNL.0000000000000667, PMID: 24991033 PMC4142003

[ref12] Braak H. Braak E. (1991). Neuropathological stageing of Alzheimer-related changes. Acta Neuropathol. 82, 239–259. doi: 10.1007/BF00308809, PMID: 1759558

[ref13] Buciuc M. Duffy J. R. Machulda M. M. Spychalla A. J. Gunter J. L. Jack C. R. Jr.. (2021). Association of amyloid angiopathy with microbleeds in logopenic progressive aphasia: an imaging-pathology study. Eur. J. Neurol. 28, 670–675. doi: 10.1111/ene.14594, PMID: 33068458 PMC8174551

[ref14] Capizzano A. A. Acion L. Bekinschtein T. Furman M. Gomila H. Martinez A.. (2004). White matter hyperintensities are significantly associated with cortical atrophy in Alzheimer's disease. J. Neurol. Neurosurg. Psychiatry 75, 822–827. doi: 10.1136/jnnp.2003.019273, PMID: 15145992 PMC1739041

[ref15] Carlos A. F. Tosakulwong N. Weigand S. D. Boeve B. F. Knopman D. S. Petersen R. C.. (2022). Frequency and distribution of TAR DNA-binding protein 43 (TDP-43) pathology increase linearly with age in a large cohort of older adults with and without dementia. Acta Neuropathol. 144, 159–160. doi: 10.1007/s00401-022-02434-3, PMID: 35536384 PMC9943023

[ref16] Carlos A. F. Tosakulwong N. Weigand S. D. Senjem M. L. Schwarz C. G. Knopman D. S.. (2023). TDP-43 pathology effect on volume and flortaucipir uptake in Alzheimer's disease. Alzheimers Dement. 19, 2343–2354. doi: 10.1002/alz.12878, PMID: 36463537 PMC10239529

[ref17] Carotenuto A. Rea R. Colucci L. Ziello A. R. Molino I. Carpi S.. (2012). Late and early onset dementia: what is the role of vascular factors? A retrospective study. J Neurol Sci 322, 170–175. doi: 10.1016/j.jns.2012.07.066, PMID: 22967745

[ref18] Chen T. B. Lee W. J. Chen J. P. Chang S. Y. Lin C. F. Chen H. C. (2022). Imaging markers of cerebral amyloid angiopathy and hypertensive arteriopathy differentiate Alzheimer disease subtypes synergistically. Alzheimers Res. Ther. 14:141. doi: 10.1186/s13195-022-01083-8, PMID: 36180874 PMC9524061

[ref19] Chen Y. Sillaire A. R. Dallongeville J. Skrobala E. Wallon D. Dubois B.. (2017). Low prevalence and clinical effect of vascular risk factors in early-onset Alzheimer's disease. J. Alzheimers Dis. 60, 1045–1054. doi: 10.3233/JAD-170367, PMID: 28984595 PMC5676853

[ref20] Corriveau-Lecavalier N. Barnard L. R. Lee J. Dicks E. Botha H. Graff-Radford J.. (2023). Deciphering the clinico-radiological heterogeneity of dysexecutive Alzheimer's disease. Cereb. Cortex 33, 7026–7043. doi: 10.1093/cercor/bhad017, PMID: 36721911 PMC10233237

[ref21] Crutch S. J. Lehmann M. Warren J. D. Rohrer J. D. (2013). The language profile of posterior cortical atrophy. J. Neurol. Neurosurg. Psychiatry 84, 460–466. doi: 10.1136/jnnp-2012-303309, PMID: 23138762 PMC4667396

[ref22] Crutch S. J. Schott J. M. Rabinovici G. D. Murray M. Snowden J. S. van der Flier W. M.. (2017). Consensus classification of posterior cortical atrophy. Alzheimers Dement. 13, 870–884. doi: 10.1016/j.jalz.2017.01.014, PMID: 28259709 PMC5788455

[ref23] Davidson Y. S. Raby S. Foulds P. G. Robinson A. Thompson J. C. Sikkink S.. (2011). TDP-43 pathological changes in early onset familial and sporadic Alzheimer's disease, late onset Alzheimer's disease and Down's syndrome: association with age, hippocampal sclerosis and clinical phenotype. Acta Neuropathol. 122, 703–713. doi: 10.1007/s00401-011-0879-y, PMID: 21968532

[ref24] Dubois B. Villain N. Frisoni G. B. Rabinovici G. D. Sabbagh M. Cappa S.. (2021). Clinical diagnosis of Alzheimer's disease: recommendations of the international working group. Lancet Neurol. 20, 484–496. doi: 10.1016/S1474-4422(21)00066-1, PMID: 33933186 PMC8339877

[ref25] Fitzpatrick D. Blanco-Campal A. Kyne L. (2019). A case of overlap posterior cortical atrophy and Logopenic variant primary progressive aphasia. Neurologist 24, 62–65. doi: 10.1097/NRL.0000000000000225, PMID: 30817493

[ref26] Forman M. S. Farmer J. Johnson J. K. Clark C. M. Arnold S. E. Coslett H. B.. (2006). Frontotemporal dementia: clinicopathological correlations. Ann. Neurol. 59, 952–962. doi: 10.1002/ana.20873, PMID: 16718704 PMC2629792

[ref27] Fox N. C. Freeborough P. A. Rossor M. N. (1996). Visualisation and quantification of rates of atrophy in Alzheimer's disease. Lancet 348, 94–97. doi: 10.1016/S0140-6736(96)05228-2, PMID: 8676724

[ref28] Foxe D. Irish M. Hu A. Carrick J. Hodges J. R. Ahmed R. M.. (2021). Longitudinal cognitive and functional changes in primary progressive aphasia. J. Neurol. 268, 1951–1961. doi: 10.1007/s00415-020-10382-9, PMID: 33417000

[ref29] Galton C. J. Patterson K. Xuereb J. H. Hodges J. R. (2000). Atypical and typical presentations of Alzheimer's disease: a clinical, neuropsychological, neuroimaging and pathological study of 13 cases. Brain 123, 484–498. doi: 10.1093/brain/123.3.484, PMID: 10686172

[ref30] Gefen T. Gasho K. Rademaker A. Lalehzari M. Weintraub S. Rogalski E.. (2012). Clinically concordant variations of Alzheimer pathology in aphasic versus amnestic dementia. Brain 135, 1554–1565. doi: 10.1093/brain/aws076, PMID: 22522938 PMC3338929

[ref31] Glenner G. G. Wong C. W. (1984). Alzheimer's disease and Down's syndrome: sharing of a unique cerebrovascular amyloid fibril protein. Biochem. Biophys. Res. Commun. 122, 1131–1135. doi: 10.1016/0006-291X(84)91209-9, PMID: 6236805

[ref32] Gorno-Tempini M. L. Dronkers N. F. Rankin K. P. Ogar J. M. Phengrasamy L. Rosen H. J.. (2004). Cognition and anatomy in three variants of primary progressive aphasia. Ann. Neurol. 55, 335–346. doi: 10.1002/ana.10825, PMID: 14991811 PMC2362399

[ref33] Gorno-Tempini M. L. Hillis A. E. Weintraub S. Kertesz A. Mendez M. Cappa S. F.. (2011). Classification of primary progressive aphasia and its variants. Neurology 76, 1006–1014. doi: 10.1212/WNL.0b013e31821103e6, PMID: 21325651 PMC3059138

[ref34] Graff-Radford J. Yong K. X. X. Apostolova L. G. Bouwman F. H. Carrillo M. Dickerson B. C.. (2021). New insights into atypical Alzheimer's disease in the era of biomarkers. Lancet Neurol. 20, 222–234. doi: 10.1016/S1474-4422(20)30440-3, PMID: 33609479 PMC8056394

[ref35] Green J. Morris J. C. Sandson J. McKeel D. W. Jr. Miller J. W. (1990). Progressive aphasia: a precursor of global dementia? Neurology 40, 423–429. doi: 10.1212/WNL.40.3_Part_1.423 2314582

[ref36] Grundke-Iqbal I. Iqbal K. Quinlan M. Tung Y. C. Zaidi M. S. Wisniewski H. M. (1986). Microtubule-associated protein tau. A component of Alzheimer paired helical filaments. J. Biol. Chem. 261, 6084–6089. doi: 10.1016/S0021-9258(17)38495-8, PMID: 3084478

[ref37] Hainsworth A. H. Markus H. S. Schneider J. A. (2023). Cerebral small vessel disease, hypertension, and vascular contributions to cognitive impairment and dementia: dementia series. Hypertension 81, 75–86. doi: 10.1161/HYPERTENSIONAHA.123.19943 38044814 PMC10734789

[ref38] Hof P. R. Bouras C. Constantinidis J. Morrison J. H. (1989). Balint's syndrome in Alzheimer's disease: specific disruption of the occipito-parietal visual pathway. Brain Res. 493, 368–375. doi: 10.1016/0006-8993(89)91173-6, PMID: 2765903

[ref39] Hu W. T. Rippon G. W. Boeve B. F. Knopman D. S. Petersen R. C. Parisi J. E.. (2009). Alzheimer's disease and corticobasal degeneration presenting as corticobasal syndrome. Mov. Disord. 24, 1375–1379. doi: 10.1002/mds.22574 19425061

[ref40] Ikeda M. Kodaira S. Kasahara H. Takai E. Nagashima K. Fujita Y.. (2021). Cerebral microbleeds, cerebrospinal fluid, and neuroimaging markers in clinical subtypes of Alzheimer's disease. Front. Neurol. 12:543866. doi: 10.3389/fneur.2021.543866, PMID: 33889121 PMC8056016

[ref41] Jack C. R. Jr. Bennett D. A. Blennow K. Carrillo M. C. Dunn B. Haeberlein S. B.. (2018). NIA-AA research framework: toward a biological definition of Alzheimer's disease. Alzheimers Dement. 14, 535–562. doi: 10.1016/j.jalz.2018.02.018, PMID: 29653606 PMC5958625

[ref42] Jack C. R. Jr. Petersen R. C. O'Brien P. C. Tangalos E. G. (1992). MR-based hippocampal volumetry in the diagnosis of Alzheimer's disease. Neurology 42, 183–188. doi: 10.1212/WNL.42.1.183, PMID: 1734300

[ref43] Johnson J. K. Head E. Kim R. Starr A. Cotman C. W. (1999). Clinical and pathological evidence for a frontal variant of Alzheimer disease. Arch. Neurol. 56, 1233–1239. doi: 10.1001/archneur.56.10.1233 10520939

[ref44] Josephs K. A. Dickson D. W. Murray M. E. Senjem M. L. Parisi J. E. Petersen R. C.. (2013). Quantitative neurofibrillary tangle density and brain volumetric MRI analyses in Alzheimer's disease presenting as logopenic progressive aphasia. Brain Lang. 127, 127–134. doi: 10.1016/j.bandl.2013.02.003, PMID: 23541297 PMC3840097

[ref45] Josephs K. A. Dickson D. W. Tosakulwong N. Weigand S. D. Murray M. E. Petrucelli L.. (2017). Rates of hippocampal atrophy and presence of post-mortem TDP-43 in patients with Alzheimer's disease: a longitudinal retrospective study. Lancet Neurol. 16, 917–924. doi: 10.1016/S1474-4422(17)30284-3, PMID: 28919059 PMC5646369

[ref46] Josephs K. A. Martin P. R. Weigand S. D. Tosakulwong N. Buciuc M. Murray M. E.. (2020a). Protein contributions to brain atrophy acceleration in Alzheimer's disease and primary age-related tauopathy. Brain 143, 3463–3476. doi: 10.1093/brain/awaa299, PMID: 33150361 PMC7719030

[ref47] Josephs K. A. Murray M. E. Whitwell J. L. Parisi J. E. Petrucelli L. Jack C. R.. (2014a). Staging TDP-43 pathology in Alzheimer's disease. Acta Neuropathol. 127, 441–450. doi: 10.1007/s00401-013-1211-9, PMID: 24240737 PMC3944799

[ref48] Josephs K. A. Murray M. E. Whitwell J. L. Tosakulwong N. Weigand S. D. Petrucelli L.. (2016). Updated TDP-43 in Alzheimer's disease staging scheme. Acta Neuropathol. 131, 571–585. doi: 10.1007/s00401-016-1537-1, PMID: 26810071 PMC5946692

[ref49] Josephs K. A. Tosakulwong N. Graff-Radford J. Weigand S. D. Buciuc M. Machulda M. M.. (2020b). MRI and flortaucipir relationships in Alzheimer's phenotypes are heterogeneous. Ann. Clin. Transl. Neurol. 7, 707–721. doi: 10.1002/acn3.51038, PMID: 32293805 PMC7261766

[ref50] Josephs K. A. Whitwell J. L. Boeve B. F. Knopman D. S. Petersen R. C. Hu W. T.. (2010). Anatomical differences between CBS-corticobasal degeneration and CBS-Alzheimer's disease. Mov. Disord. 25, 1246–1252. doi: 10.1002/mds.23062, PMID: 20629131 PMC2921765

[ref51] Josephs K. A. Whitwell J. L. Knopman D. S. Hu W. T. Stroh D. A. Baker M.. (2008). Abnormal TDP-43 immunoreactivity in AD modifies clinicopathologic and radiologic phenotype. Neurology 70, 1850–1857. doi: 10.1212/01.wnl.0000304041.09418.b1, PMID: 18401022 PMC2779031

[ref52] Josephs K. A. Whitwell J. L. Tosakulwong N. Weigand S. D. Murray M. E. Liesinger A. M.. (2015). TAR DNA-binding protein 43 and pathological subtype of Alzheimer's disease impact clinical features. Ann. Neurol. 78, 697–709. doi: 10.1002/ana.24493 26224156 PMC4623932

[ref53] Josephs K. A. Whitwell J. L. Weigand S. D. Murray M. E. Tosakulwong N. Liesinger A. M.. (2014b). TDP-43 is a key player in the clinical features associated with Alzheimer's disease. Acta Neuropathol. 127, 811–824. doi: 10.1007/s00401-014-1269-z, PMID: 24659241 PMC4172544

[ref54] Joubert S. Gour N. Guedj E. Didic M. Guériot C. Koric L.. (2016). Early-onset and late-onset Alzheimer's disease are associated with distinct patterns of memory impairment. Cortex 74, 217–232. doi: 10.1016/j.cortex.2015.10.014 26694580

[ref55] Kanne S. M. Balota D. A. Storandt M. McKeel D. W. Jr. Morris J. C. (1998). Relating anatomy to function in Alzheimer's disease: neuropsychological profiles predict regional neuropathology 5 years later. Neurology 50, 979–985. doi: 10.1212/WNL.50.4.979 9566382

[ref56] Katzman R. (1976). Editorial: the prevalence and malignancy of Alzheimer disease. A major killer. Arch. Neurol. 33, 217–218. doi: 10.1001/archneur.1976.00500040001001 1259639

[ref57] Kertesz A. McMonagle P. Blair M. Davidson W. Munoz D. G. (2005). The evolution and pathology of frontotemporal dementia. Brain 128, 1996–2005. doi: 10.1093/brain/awh598 16033782

[ref58] Kidd M. (1963). Paired helical filaments in electron microscopy of Alzheimer's disease. Nature 197, 192–193. doi: 10.1038/197192b0 14032480

[ref59] Koedam E. L. Lauffer V. van der Vlies A. E. van der Flier W. M. Scheltens P. Pijnenburg Y. A. (2010). Early-versus late-onset Alzheimer's disease: more than age alone. J. Alzheimers Dis. 19, 1401–1408. doi: 10.3233/JAD-2010-1337, PMID: 20061618

[ref60] Koga S. Josephs K. A. Aiba I. Yoshida M. Dickson D. W. (2022). Neuropathology and emerging biomarkers in corticobasal syndrome. J. Neurol. Neurosurg. Psychiatry 93, 919–929. doi: 10.1136/jnnp-2021-328586, PMID: 35697501 PMC9380481

[ref61] La Joie R. Visani A. V. Lesman-Segev O. H. Baker L. Edwards L. Iaccarino L.. (2021). Association of APOE4 and clinical variability in Alzheimer disease with the pattern of tau- and amyloid-PET. Neurology 96, e650–e661. doi: 10.1212/WNL.0000000000011270, PMID: 33262228 PMC7884991

[ref62] Launer L. J. Petrovitch H. Ross G. W. Markesbery W. White L. R. (2008). AD brain pathology: vascular origins? Results from the HAAS autopsy study. Neurobiol. Aging 29, 1587–1590. doi: 10.1016/j.neurobiolaging.2007.03.008, PMID: 17466414 PMC3437222

[ref63] Lee S. E. Rabinovici G. D. Mayo M. C. Wilson S. M. Seeley W. W. DeArmond S. J.. (2011). Clinicopathological correlations in corticobasal degeneration. Ann. Neurol. 70, 327–340. doi: 10.1002/ana.22424, PMID: 21823158 PMC3154081

[ref64] Levine D. N. Lee J. M. Fisher C. M. (1993). The visual variant of Alzheimer's disease: a clinicopathologic case study. Neurology 43, 305–313. doi: 10.1212/WNL.43.2.305 8437694

[ref65] Ling H. O'Sullivan S. S. Holton J. L. Revesz T. Massey L. A. Williams D. R.. (2010). Does corticobasal degeneration exist? A clinicopathological re-evaluation. Brain 133, 2045–2057. doi: 10.1093/brain/awq123, PMID: 20584946

[ref66] Logue M. W. Posner H. Green R. C. Moline M. Cupples L. A. Lunetta K. L.. (2011). Magnetic resonance imaging-measured atrophy and its relationship to cognitive functioning in vascular dementia and Alzheimer's disease patients. Alzheimers Dement. 7, 493–500. doi: 10.1016/j.jalz.2011.01.004, PMID: 21723205 PMC3166967

[ref67] Longstreth W. T. Jr. Manolio T. A. Arnold A. Burke L. Bryan N. Jungreis C. A.. (1996). Clinical correlates of white matter findings on cranial magnetic resonance imaging of 3301 elderly people. The Cardiovascular Health Study. Stroke 27, 1274–1282. doi: 10.1161/01.STR.27.8.1274 8711786

[ref68] Machulda M. M. Whitwell J. L. Duffy J. R. Strand E. A. Dean P. M. Senjem M. L.. (2013). Identification of an atypical variant of logopenic progressive aphasia. Brain Lang. 127, 139–144. doi: 10.1016/j.bandl.2013.02.007 23566690 PMC3725183

[ref69] Martersteck A. Ayala I. Ohm D. T. Spencer C. Coventry C. Weintraub S.. (2022). Focal amyloid and asymmetric tau in an imaging-to-autopsy case of clinical primary progressive aphasia with Alzheimer disease neuropathology. Acta Neuropathol. Commun. 10:111. doi: 10.1186/s40478-022-01412-w, PMID: 35945628 PMC9361632

[ref70] McKhann G. Drachman D. Folstein M. Katzman R. Price D. Stadlan E. M. (1984). Clinical diagnosis of Alzheimer's disease: report of the NINCDS-ADRDA work group under the auspices of Department of Health and Human Services Task Force on Alzheimer's disease. Neurology 34, 939–944. doi: 10.1212/WNL.34.7.939 6610841

[ref71] McKhann G. M. Knopman D. S. Chertkow H. Hyman B. T. Jack C. A. Hawas C. H.. (2011). The diagnosis of dementia due to Alzheimer's disease: recommendations from the National Institute on Aging-Alzheimer's Association workgroups on diagnostic guidelines for Alzheimer's disease. Alzheimers Dement. 7, 263–269. doi: 10.1016/j.jalz.2011.03.005, PMID: 21514250 PMC3312024

[ref72] Mendes A. Bertrand A. Lamari F. Colliot O. Routier A. Godefroy O.. (2018). Cerebral microbleeds and CSF Alzheimer biomarkers in primary progressive aphasias. Neurology 90, e1057–e1065. doi: 10.1212/WNL.0000000000005165 29444966

[ref73] Mendez M. F. Monserratt L. H. Liang L. J. Chavez D. Jimenez E. E. Maurer J. J.. (2019). Neuropsychological similarities and differences between amnestic Alzheimer's disease and its non-amnestic variants. J. Alzheimers Dis. 69, 849–855. doi: 10.3233/JAD-190124, PMID: 31156165

[ref74] Mesulam M. Wicklund A. Johnson N. Rogalski E. Léger G. C. Rademaker A.. (2008). Alzheimer and frontotemporal pathology in subsets of primary progressive aphasia. Ann. Neurol. 63, 709–719. doi: 10.1002/ana.21388, PMID: 18412267 PMC2858311

[ref75] Murray M. E. Graff-Radford N. R. Ross O. A. Petersen R. C. Duara R. Dickson D. W. (2011). Neuropathologically defined subtypes of Alzheimer's disease with distinct clinical characteristics: a retrospective study. Lancet Neurol. 10, 785–796. doi: 10.1016/S1474-4422(11)70156-9, PMID: 21802369 PMC3175379

[ref76] Neary D. Snowden J. S. Bowen D. M. Sims N. R. Mann D. M. Benton J. S.. (1986). Neuropsychological syndromes in presenile dementia due to cerebral atrophy. J. Neurol. Neurosurg. Psychiatry 49, 163–174. doi: 10.1136/jnnp.49.2.163, PMID: 2419511 PMC1028682

[ref77] Neumann M. Sampathu D. M. Kwong L. K. Truax A. C. Micsenyi M. C. Chou T. T.. (2006). Ubiquitinated TDP-43 in frontotemporal lobar degeneration and amyotrophic lateral sclerosis. Science 314, 130–133. doi: 10.1126/science.1134108 17023659

[ref78] Ossenkoppele R. Pijnenburg Y. A. Perry D. C. Cohn-Sheehy B. I. Scheltens N. M. Vogel J. W.. (2015). The behavioural/dysexecutive variant of Alzheimer's disease: clinical, neuroimaging and pathological features. Brain 138, 2732–2749. doi: 10.1093/brain/awv191, PMID: 26141491 PMC4623840

[ref79] Ossenkoppele R. Schonhaut D. R. Scholl M. Lockhart S. N. Ayakta N. Baker S. L.. (2016). Tau PET patterns mirror clinical and neuroanatomical variability in Alzheimer's disease. Brain 139, 1551–1567. doi: 10.1093/brain/aww027 26962052 PMC5006248

[ref80] Ossenkoppele R. Singleton E. H. Groot C. Dijkstra A. A. Eikelboom W. S. Seeley W. W.. (2022). Research criteria for the behavioral variant of Alzheimer disease: a systematic review and Meta-analysis. JAMA Neurol. 79, 48–60. doi: 10.1001/jamaneurol.2021.4417, PMID: 34870696 PMC8649917

[ref81] Ouchi H. Toyoshima Y. Tada M. Oyake M. Aida I. Tomita I.. (2014). Pathology and sensitivity of current clinical criteria in corticobasal syndrome. Mov. Disord. 29, 238–244. doi: 10.1002/mds.25746, PMID: 24259271

[ref82] Owens T. E. Machulda M. M. Duffy J. R. Strand E. A. Clark H. M. Boland S.. (2018). Patterns of neuropsychological dysfunction and cortical volume changes in Logopenic aphasia. J. Alzheimers Dis. 66, 1015–1025. doi: 10.3233/JAD-171175, PMID: 30372673 PMC6322407

[ref83] Petersen C. Nolan A. L. De Paula Franca Resende E. Miller Z. Ehrenberg A. J. Gorno-Tempini M. L.. (2019). Alzheimer's disease clinical variants show distinct regional patterns of neurofibrillary tangle accumulation. Acta Neuropathol. 138, 597–612. doi: 10.1007/s00401-019-02036-6 31250152 PMC7012374

[ref84] Pham N. T. T. Graff-Radford J. Machulda M. M. Spychalla A. J. Schwarz C. G. Senjem M. L.. (2022). Regional white matter hyperintensities in posterior cortical atrophy and logopenic progressive aphasia. Neurobiol. Aging 119, 46–55. doi: 10.1016/j.neurobiolaging.2022.07.008, PMID: 35970009 PMC9886198

[ref85] Phillips J. S. Da Re F. Dratch L. Xie S. X. Irwin D. J. McMillan C. T.. (2018). Neocortical origin and progression of gray matter atrophy in nonamnestic Alzheimer's disease. Neurobiol. Aging 63, 75–87. doi: 10.1016/j.neurobiolaging.2017.11.008, PMID: 29223682 PMC5801003

[ref86] Pillai J. A. Bonner-Jackson A. Bekris L. M. Safar J. Bena J. Leverenz J. B. (2019). Highly elevated cerebrospinal fluid Total tau level reflects higher likelihood of non-amnestic subtype of Alzheimer's disease. J. Alzheimers Dis. 70, 1051–1058. doi: 10.3233/JAD-190519, PMID: 31306137 PMC7086408

[ref87] Pogacar S. Williams R. S. (1976). Alzheimer's disease presenting as slowly progressive aphasia. R I Med. J. 1984, 181–185. 6587514

[ref88] Price B. H. Gurvit H. Weintraub S. Geula C. Leimkuhler E. Mesulam M. (1993). Neuropsychological patterns and language deficits in 20 consecutive cases of autopsy-confirmed Alzheimer's disease. Arch. Neurol. 50, 931–937. doi: 10.1001/archneur.1993.00540090038008, PMID: 8363447

[ref89] Rogalski E. Sridhar J. Rader B. Martersteck A. Chen K. Cobia D.. (2016). Aphasic variant of Alzheimer disease: clinical, anatomic, and genetic features. Neurology 87, 1337–1343. doi: 10.1212/WNL.0000000000003165, PMID: 27566743 PMC5047036

[ref90] Ross S. J. Graham N. Stuart-Green L. Prins M. Xuereb J. Patterson K.. (1996). Progressive biparietal atrophy: an atypical presentation of Alzheimer's disease. J. Neurol. Neurosurg. Psychiatry 61, 388–395. doi: 10.1136/jnnp.61.4.388, PMID: 8890778 PMC486580

[ref91] Sahoo A. Bejanin A. Murray M. E. Tosakulwong N. Weigand S. D. Serie A. M.. (2018). TDP-43 and Alzheimer's disease pathologic subtype in non-amnestic Alzheimer's disease dementia. J. Alzheimers Dis. 64, 1227–1233. doi: 10.3233/JAD-180169, PMID: 30010126 PMC6258191

[ref92] Sarto J. Maya G. Molina-Porcel L. Balasa M. Gelpi E. Aldecoa I.. (2022). Evolution of clinical-pathological correlations in early-onset Alzheimer's disease over a 25-year period in an academic brain Bank. J. Alzheimers Dis. 87, 1659–1669. doi: 10.3233/JAD-220045 35723108

[ref93] Scheltens P. Barkhof F. Valk J. Algra P. R. van der Hoop R. G. Nauta J.. (1992). White matter lesions on magnetic resonance imaging in clinically diagnosed Alzheimer's disease. Evidence for heterogeneity. Brain 115, 735–748. doi: 10.1093/brain/115.3.735 1628199

[ref94] Seab J. P. Jagust W. J. Wong S. T. Roos M. S. Reed B. R. Budinger T. F. (1988). Quantitative NMR measurements of hippocampal atrophy in Alzheimer's disease. Magn. Reson. Med. 8, 200–208. doi: 10.1002/mrm.1910080210 3210957

[ref95] Shelley B. P. Hodges J. R. Kipps C. M. Xuereb J. H. Bak T. H. (2009). Is the pathology of corticobasal syndrome predictable in life? Mov. Disord. 24, 1593–1599. doi: 10.1002/mds.22558, PMID: 19533751

[ref96] Shir D. Pham N. T. T. Botha H. Koga S. Kouri N. Ahlskog J. E.. (2023). Clinico-radiologic and pathological evaluation of Corticobasal syndrome. Neurology. doi: 10.1212/WNL.0000000000207397 PMC10382268 37268436

[ref97] Singh N. A. Graff-Radford J. Machulda M. M. Schwarz C. G. Baker M. C. Rademakers R.. (2022). Atypical Alzheimer's disease phenotypes with normal or borderline PET biomarker profiles. J. Neurol. 269, 6613–6626. doi: 10.1007/s00415-022-11330-5, PMID: 36001141 PMC9707302

[ref98] Singh N. A. Graff-Radford J. Machulda M. Carlos A. F. Schwarz C. G. Senjem M. L.. (2023). Atypical Alzheimer’s disease: new insights into an overlapping spectrum between the language and visual variants. Alzheimer's Association international conference. Alzheimer's & Dementia 19, 658–670. doi: 10.1002/alz.079058 PMC11273322 38551740

[ref99] Singleton E. H. Pijnenburg Y. A. L. Sudre C. H. Groot C. Kochova E. Barkhof F.. (2020). Investigating the clinico-anatomical dissociation in the behavioral variant of Alzheimer disease. Alzheimers Res. Ther. 12:148. doi: 10.1186/s13195-020-00717-z, PMID: 33189136 PMC7666520

[ref100] Sintini I. Graff-Radford J. Senjem M. L. Schwarz C. G. Machulda M. M. Martin P. R.. (2020). Longitudinal neuroimaging biomarkers differ across Alzheimer's disease phenotypes. Brain 143, 2281–2294. doi: 10.1093/brain/awaa155, PMID: 32572464 PMC7363492

[ref101] Sintini I. Schwarz C. G. Martin P. R. Graff-Radford J. Machulda M. M. Senjem M. L.. (2019). Regional multimodal relationships between tau, hypometabolism, atrophy, and fractional anisotropy in atypical Alzheimer's disease. Hum. Brain Mapp. 40, 1618–1631. doi: 10.1002/hbm.24473, PMID: 30549156 PMC6615561

[ref102] Snowdon D. A. Greiner L. H. Mortimer J. A. Riley K. P. Greiner P. A. Markesbery W. R. (1997). Brain infarction and the clinical expression of Alzheimer disease. The Nun Study. JAMA 277, 813–817. doi: 10.1001/jama.1997.03540340047031, PMID: 9052711

[ref103] Tang-Wai D. F. Graff-Radford N. R. Boeve B. F. Dickson D. W. Parisi J. E. Crook R.. (2004). Clinical, genetic, and neuropathologic characteristics of posterior cortical atrophy. Neurology 63, 1168–1174. doi: 10.1212/01.WNL.0000140289.18472.15, PMID: 15477533

[ref104] Tanner J. A. Iaccarino L. Edwards L. Asken B. M. Gorno-Tempini M. L. Kramer J. H.. (2022). Amyloid, tau and metabolic PET correlates of cognition in early and late-onset Alzheimer's disease. Brain 145, 4489–4505. doi: 10.1093/brain/awac229, PMID: 35762829 PMC10200306

[ref105] Tee B. L. Watson Pereira C. Lukic S. Bajorek L. P. Allen I. E. Miller Z. A.. (2022). Neuroanatomical correlations of visuospatial processing in primary progressive aphasia. Brain Commun 4:fcac060. doi: 10.1093/braincomms/fcac060, PMID: 35386217 PMC8977647

[ref106] Tetzloff K. A. Duffy J. R. Strand E. A. Machulda M. M. Schwarz C. G. Senjem M. L.. (2021). Phonological errors in posterior cortical atrophy. Dement. Geriatr. Cogn. Disord. 50, 195–203. doi: 10.1159/000516481, PMID: 34274933 PMC8376759

[ref107] Therriault J. Pascoal T. A. Savard M. Benedet A. L. Chamoun M. Tissot C.. (2021). Topographic distribution of amyloid-beta, tau, and atrophy in patients with behavioral/Dysexecutive Alzheimer disease. Neurology 96, e81–e92. doi: 10.1212/WNL.0000000000011081, PMID: 33093220 PMC7884976

[ref108] Tippett D. C. Breining B. Goldberg E. Meier E. Sheppard S. M. Sherry E.. (2020). Visuomotor Figure construction and visual Figure delayed recall and recognition in primary progressive aphasia. Aphasiology 34, 1456–1470. doi: 10.1080/02687038.2019.1670330, PMID: 33281269 PMC7716596

[ref109] Tort-Merino A. Falgas N. Allen I. E. Balasa M. Olives J. Cantador J.. (2022). Early-onset Alzheimer's disease shows a distinct neuropsychological profile and more aggressive trajectories of cognitive decline than late-onset. Ann. Clin. Transl. Neurol. 9, 1962–1973. doi: 10.1002/acn3.51689, PMID: 36398437 PMC9735361

[ref110] Townley R. A. Graff-Radford J. Mantyh W. G. Botha H. Polsinelli A. J. Przybelski S. A.. (2020). Progressive dysexecutive syndrome due to Alzheimer's disease: a description of 55 cases and comparison to other phenotypes. Brain Commun. 2:fcaa068. doi: 10.1093/braincomms/fcaa068, PMID: 32671341 PMC7325839

[ref111] Uryu K. Nakashima-Yasuda H. Forman M. S. Kwong L. K. Clark C. M. Grossman M.. (2008). Concomitant TAR-DNA-binding protein 43 pathology is present in Alzheimer disease and corticobasal degeneration but not in other tauopathies. J. Neuropathol. Exp. Neurol. 67, 555–564. doi: 10.1097/NEN.0b013e31817713b5, PMID: 18520774 PMC3659339

[ref112] Vernooij M. W. van der Lugt A. Ikram M. A. Wielopolski P. A. Niessen W. J. Hofman A.. (2008). Prevalence and risk factors of cerebral microbleeds: the Rotterdam scan study. Neurology 70, 1208–1214. doi: 10.1212/01.wnl.0000307750.41970.d9 18378884

[ref113] Volkmer A. Farrington-Douglas C. Crutch S. Beeke S. Warren J. Yong K. (2022). Better conversations: a language and communication intervention for aphasia in posterior cortical atrophy. Neurocase 28, 356–363. doi: 10.1080/13554794.2022.2125326, PMID: 36130333 PMC9612924

[ref114] Wellington H. Paterson R. W. Suarez-Gonzalez A. Poole T. Frost C. Sjobom U.. (2018). CSF neurogranin or tau distinguish typical and atypical Alzheimer disease. Ann. Clin. Transl. Neurol. 5, 162–171. doi: 10.1002/acn3.518, PMID: 29468177 PMC5817822

[ref115] Whitwell J. L. Graff-Radford J. Tosakulwong N. Weigand S. D. Machulda M. Senjem M. L.. (2018a). [(18) F]AV-1451 clustering of entorhinal and cortical uptake in Alzheimer's disease. Ann. Neurol. 83, 248–257. doi: 10.1002/ana.25142, PMID: 29323751 PMC5821532

[ref116] Whitwell J. L. Graff-Radford J. Tosakulwong N. Weigand S. D. Machulda M. M. Senjem M. L.. (2018b). Imaging correlations of tau, amyloid, metabolism, and atrophy in typical and atypical Alzheimer's disease. Alzheimers Dement. 14, 1005–1014. doi: 10.1016/j.jalz.2018.02.020, PMID: 29605222 PMC6097955

[ref117] Whitwell J. L. Jack C. R. Jr. Duffy J. R. Strand E. A. Gunter J. L. Senjem M. L.. (2014). Microbleeds in the logopenic variant of primary progressive aphasia. Alzheimers Dement. 10, 62–66. doi: 10.1016/j.jalz.2013.01.006, PMID: 23562427 PMC3706560

[ref118] Whitwell J. L. Jack C. R. Jr. Kantarci K. Weigand S. D. Boeve B. F. Knopman D. S.. (2007). Imaging correlates of posterior cortical atrophy. Neurobiol. Aging 28, 1051–1061. doi: 10.1016/j.neurobiolaging.2006.05.026, PMID: 16797786 PMC2734142

[ref119] Whitwell J. L. Jack C. R. Jr. Przybelski S. A. Parisi J. E. Senjem M. L. Boeve B. F.. (2011). Temporoparietal atrophy: a marker of AD pathology independent of clinical diagnosis. Neurobiol. Aging 32, 1531–1541. doi: 10.1016/j.neurobiolaging.2009.10.012, PMID: 19914744 PMC2888989

[ref120] Whitwell J. L. Kantarci K. Weigand S. D. Lundt E. S. Gunter J. L. Duffy J. R.. (2015). Microbleeds in atypical presentations of Alzheimer's disease: a comparison to dementia of the Alzheimer's type. J. Alzheimers Dis. 45, 1109–1117. doi: 10.3233/JAD-142628, PMID: 25649655 PMC5540348

[ref121] Whitwell J. L. Martin P. Graff-Radford J. Machulda M. M. Senjem M. L. Schwarz C. G.. (2019). The role of age on tau PET uptake and gray matter atrophy in atypical Alzheimer's disease. Alzheimers Dement. 15, 675–685. doi: 10.1016/j.jalz.2018.12.016, PMID: 30853465 PMC6511453

[ref122] Whitwell J. Martin P. R. Graff-Radford J. Machulda M. M. Sintini I. Buciuc M.. (2022). Investigating heterogeneity and neuroanatomic correlates of longitudinal clinical decline in atypical Alzheimer disease. Neurology 98, e2436–e2445. doi: 10.1212/WNL.0000000000200336, PMID: 35483899 PMC9231842

[ref123] Wicklund M. R. Duffy J. R. Strand E. A. Whitwell J. L. Machulda M. M. Josephs K. A. (2013). Aphasia with left occipitotemporal hypometabolism: a novel presentation of posterior cortical atrophy? J. Clin. Neurosci. 20, 1237–1240. doi: 10.1016/j.jocn.2013.01.002, PMID: 23850398 PMC4217166

[ref124] Wilson R. S. Yu L. Trojanowski J. Q. Chen E. Y. Boyle P. A. Bennett D. A.. (2013). TDP-43 pathology, cognitive decline, and dementia in old age. JAMA Neurol. 70, 1418–1424. doi: 10.1001/jamaneurol.2013.3961, PMID: 24080705 PMC3830649

[ref125] Xia C. Makaretz S. J. Caso C. McGinnis S. Gomperts S. N. Sepulcre J.. (2017). Association of *in vivo* [18F]AV-1451 tau PET imaging results with cortical atrophy and symptoms in typical and atypical Alzheimer disease. JAMA Neurol. 74, 427–436. doi: 10.1001/jamaneurol.2016.5755, PMID: 28241163 PMC5470368

